# Treatment of locally advanced rectal cancer and synchronous liver metastases: multicentre comparison of two treatment strategies

**DOI:** 10.1093/bjs/znad013

**Published:** 2023-02-23

**Authors:** Jan M van Rees, Myrtle F Krul, Niels F M Kok, Dirk J Grünhagen, E N D Kok, Pieter M H Nierop, Klaas Havenga, Harm Rutten, Jacobus W A Burger, Johannes H W de Wilt, Jeroen Hagendoorn, Femke P Peters, Johannes Buijsen, Pieter J Tanis, Cornelis Verhoef, Koert F D Kuhlmann, Dutch Stage IV Rectal Cancer Group, G L Beets, A G J Aalbers, T J M Ruers, C B H A Kobus, S V Siemons, C Grootscholten, L G H Dewit, J G van den Berg, K P de Jong, G A P Hospers, A Karrenbeld, E D Geijsen, C J A Punt, E Gootjes, M P W Intven, J M L Roodhart, F Holman, E Kapiteijn, J Melenhorst, J S Cnossen, G J M Creemers

**Affiliations:** Department of Surgical Oncology, Erasmus MC Cancer Institute, Rotterdam, the Netherlands; Department of Surgical Oncology, Netherlands Cancer Institute, Amsterdam, the Netherlands; Department of Surgical Oncology, Netherlands Cancer Institute, Amsterdam, the Netherlands; Department of Surgical Oncology, Erasmus MC Cancer Institute, Rotterdam, the Netherlands; Department of Surgical Oncology, Netherlands Cancer Institute, Amsterdam, the Netherlands; Department of Surgical Oncology, Erasmus MC Cancer Institute, Rotterdam, the Netherlands; Department of Surgery, University of Groningen, Groningen, the Netherlands; Department of Surgery, Catharina Hospital, Eindhoven, the Netherlands; Department of Surgery, Catharina Hospital, Eindhoven, the Netherlands; Department of Surgery, Radboud University Medical Centre, Nijmegen, the Netherlands; Department of Surgery, University Medical Centre Utrecht, Utrecht, the Netherlands; Department of Radiation Oncology, Leiden University Medical Centre, Leiden, the Netherlands; Department of Radiation Oncology (Maastro), GROW School for Oncology, Maastricht University Medical Centre+, Maastricht, the Netherlands; Department of Surgical Oncology, Erasmus MC Cancer Institute, Rotterdam, the Netherlands; Department of Surgery, Amsterdam UMC, Cancer Centre Amsterdam, University of Amsterdam, Amsterdam, the Netherlands; Department of Surgical Oncology, Erasmus MC Cancer Institute, Rotterdam, the Netherlands; Department of Surgical Oncology, Netherlands Cancer Institute, Amsterdam, the Netherlands

## Introduction

Management of locally advanced rectal cancer (LARC) has been optimized during recent times through (beyond) total mesorectal excision surgery and, more recently, the introduction of total neoadjuvant treatment (TNT)^1–4^. For patients with synchronous liver metastases, the optimal treatment strategy is less clear, with high variability among institutions worldwide^5^. In the Netherlands, two specific treatment sequences have mainly been used for treating LARC and synchronous liver metastases: the liver-first approach (LFA) and the M1 schedule^6–8^.

The LFA consists of induction systemic chemotherapy, subsequent local treatment of the liver metastases, followed by long-course (chemo)radiotherapy and resection of the primary tumour. The rationale behind LFA is to treat the rectal tumour locally only when control of synchronous liver metastases has been established. Radiotherapy and primary tumour resection can be avoided in patients with disease progression during the first phase of the schedule^9^.

The M1 schedule starts with preoperative short-course pelvic radiotherapy (5 × 5 Gy), followed by systemic therapy, and subsequent surgical treatment of both the liver and rectum (either simultaneously, liver first or primary tumour first). The advantage of starting with radiotherapy is the immediate downstaging effect on the primary tumour. This strategy has been proven safe and effective, and leads to excellent local control^10^.

The aim of this study was to compare outcomes between the LFA and the M1 schedule.

## Methods

This was a multicentre retrospective study including patients with LARC and synchronous liver metastases. The choice of either LFA or M1 was based on the centre in which patients were treated, and not on whether the patient experienced bleeding or obstruction. Main outcomes of interest were schedule completion, progression-free survival, overall survival, and response rates. Detailed information on the treatment schedules investigated, definitions, outcome measures, and statistical analyses are provided in the *supplementary material*.

## Results

Some 260 patients were identified, of whom 96 (37.1 per cent) and 164 (62.9 per cent) were treated according to the LFA and M1 schedule respectively (*Table 1*). Major complications related to local treatment of the liver occurred in 4 (4 per cent) and 16 (15 per cent) patients respectively (*P* = 0.010). The complication rate was particularly high in patients who underwent simultaneous resection in M1 (33 per cent), and was higher than the total complication rate of 16 per cent for staged resections in LFA (4 per cent liver and 12 per cent rectal resections). Detailed information on treatment and complications are available in the *supplementary material.*

**Table 1:** Baseline and treatment characteristics

	LFA (*n* = 96)	M1 (*n* = 164)	P*
Age (years), median (i.q.r.)	62.6 (56.3–67.8)	61.6 (55.4–68.7)	0.646†
Sex ratio (M : F)	67 : 29	116 : 48	0.873
Co-morbidity	54 (56)	85 (52)	0.490
**Clinical T category**			0.175
cT2	1 (1)	4 (2)	
cT3	57 (63)	119 (73)	
cT4	32 (36)	41 (25)	
**Clinical N category**			0.709
cN0	4 (5)	12 (7)	
cN1	22 (28)	50 (31)	
cN2	52 (67)	101 (62)	
**LM distribution**			0.102
Unilobar	42 (44)	89 (54)	
Bilobar	54 (56)	75 (46)	
No. of LMs at diagnosis (median, i.q.r.)	3 (2–5)	2 (1–4)	0.011†
Diameter of largest LM lesion at diagnosis (cm), median (i.q.r.)	2.8 (2.0–4.4)	2.8 (2.0–4.2)	0.941†
Extrahepatic disease at diagnosis	13 (14)	32 (20)	0.219
**Completion of treatment**			0.245
No	24 (25)	31 (19)	
Yes	72 (75)	133 (81)	
**LM pCR**			0.357
Yes	10 (12)	18 (17)	
No	73 (88)	89 (83)	
**Rectal complete response**			0.266
No	59 (91)	113 (88)	
pCR	6 (9)	10 (8)	
cCR	0 (0)	5 (4)	
Treatment duration if scheme completed (weeks), median (i.q.r.)	44.0 (39.5, 49.9)	35.9 (29.5, 42.6)	<0.001†
Total duration of hospital stay if scheme completed (days), (mean(s.d.)	18.8(8.9)	18.0(11.8)	0.686†

Values are *n* (%) unless otherwise indicated. LFA, liver-first approach; M1, M1 schedule; LM, liver metastasis. *χ^2^ test, except †Mann–Whitney *U* test.

For patients who completed the schedule, median treatment duration was 44.0 (i.q.r. 39.5–49.9) and 35.9 (29.5–42.6) weeks in the LFA and M1 groups respectively (*P* < 0.001). Complete responses (cCR or pCR) of the primary tumour were observed in 6 (9 per cent) and 15 (12 per cent) patients respectively (*P* = 0.266).

### Survival

**Fig. 1:**
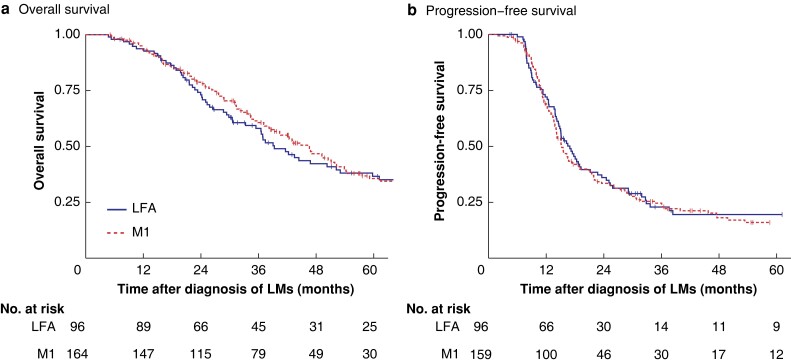
Overall and progression-free survival among patients treated with liver-first approach or M1 schedule **a** Overall and **b** progression-free survival. **a***P* = 0.209, **b***P* = 0.575 (log rank test).

Median follow-up was 33.3 and 34.6 months for the LFA and M1 groups. Survival did not differ between groups (*Fig. 1*). At the end of follow-up, the number of patients with pelvic local recurrence after schedule completion was 5 (6.9 per cent) and 13 (9.8 per cent) respectively (*P* = 0.494).

## Discussion

This study compared two accepted treatment schedules for patients with potentially curable LARC and synchronous liver metastases. Overall and progression-free survival were similar after either treatment. This is in line with several studies that compared different treatment sequences in patients with colorectal cancer and synchronous liver metastases^11–14^. M1 has not yet been compared with any other schedule. The present findings suggest that M1 and LFA have similar outcomes.

Observed complete response rates were lower than rates reported in recent TNT trials^1,15–17^. In the RAPIDO trial^15^, patients who underwent short-course radiotherapy and subsequent systemic chemotherapy had a complete response rate of 28 per cent. It has, however, been suggested that patients with larger tumours might not have experienced the same downstaging effects after TNT as those with smaller, earlier-stage tumours in the RAPIDO trial^18^. In a previous study^10^ of patients treated according to the M1 schedule for metastatic disease, the complete response rate was 26 per cent. This study also included patients without LARC. This implies that the downstaging effect of TNT can be expected to be more pronounced in patients with T2 or T3 tumours than in patients with larger, locally advanced tumours and synchronous liver metastases. The relatively small proportion of complete responders after LFA and M1 is likely to be explained by more aggressive biological disease behaviour and larger tumours.

As a watch-and-wait approach is safe in stage IV rectal cancer with a (near) complete response, future research should focus on optimal selection of patients with metastatic LARC who can be treated with organ preservation^19^. Such strategies are especially of interest in these patients as prognosis is mainly determined by metastases.

Major complications after liver surgery were more frequently observed in the M1 group. In particular, patients who underwent simultaneous resection of the primary tumour and metastases were at higher risk of complications. It should, however, be noted that, besides differences in treatment sequence, other factors such as case mix (for example preoperative chemotherapeutic regimen) could also have played an important role in morbidity outcomes. Median duration of the completed M1 schedule was 8 weeks shorter than that for LFA, roughly reflecting the difference in the duration of radiation schedules (1 week of short-course radiotherapy in M1 *versus* 5 weeks of long-course chemoradiotherapy) plus a waiting period. These factors may influence future decision-making and counselling of patients.

Both the M1 schedule and LFA have their own advantages and disadvantages. Initiating a randomized trial, however, will probably not provide additional value. Alternatively, either schedule can be preferred in individual patients. For example, in patients with symptomatic rectal cancer, such as bleeding and obstructive symptoms, upfront short-course radiotherapy can be administered to obtain durable local control, and may reduce the risk of an emergency stoma compared with the downstaging effects of systemic chemotherapy only^20^. Additionally, the interval after short-course radiotherapy can be used efficiently to treat the liver with systemic chemotherapy and surgery, while observing the local behaviour of the primary tumour when a (near) complete response is found. Patients with progressive metastatic disease during the first phase of the schedule may, however, not benefit from downstaging of the primary tumour, and radiotherapy can lead to both morbidity and futile costs. LFA might therefore be more convenient in patients with more extensive liver metastases at the time of diagnosis, in whom the chance of completion of the full schedule is expected to be lower. A downside of LFA is that simultaneous resection of both tumour sites is not possible, which can be a valuable treatment option in selected patients. To guarantee appropriate patient counselling, a multidisciplinary team with expertise in both the treatment of advanced primary (colo)rectal cancer and colorectal liver metastases is warranted. Firmly established infrastructure between local hospitals and timely referral to an expert centre is required for either of these strategies to work.

Limitations of this study included the retrospective design. Patients with progressive disease or clinical deterioration before any surgery were not included. Although the proportion of patients with disease progression during neoadjuvant treatment was likely to be similar in both treatment schedules, some selection bias was unavoidable, and (oncological) survival outcomes in this study were probably better than those achieved in daily practice on an intention-to-treat basis. The number of patients included in this study was relatively small, and differences in baseline and inclusion periods between the two treatment groups might have affected outcomes reported in this study.

## Supplementary Material

znad013_Supplementary_Data

## Data Availability

The data analysed in this study are not publicly available owing to ethical concerns. Further information about the data and conditions for access are available by contacting the corresponding author.

## Collaborators

Dutch Stage IV Rectal Cancer Group: G.L. Beets, A.G.J. Aalbers, T. J. M. Ruers, C. B. H. A. Kobus, S. V. Siemons, C. Grootscholten, L.G.H. Dewit and J.G. van den Berg (Netherlands Cancer institute, Amsterdam, Netherlands), K.P. de Jong, G.A.P. Hospers and A. Karrenbeld (University Medical Centre Groningen, Groningen, Netherlands), E.D. Geijsen and C.J.A. Punt (Amsterdam University Medical Centre, Amsterdam, Netherlands), E. Gootjes (Radboud University Medical Centre, Nijmegen, Netherlands), M.P.W. Intven and J.M.L. Roodhart (University Medical Centre Utrecht, Utrecht, Netherlands), F. Holman and E. Kapiteijn (Leiden University Medical Centre, Leiden, Netherlands), J. Melenhorst (Maastricht University Medical Centre, Maastricht, Netherlands), J.S. Cnossen and G.J.M. Creemers (Catharina Hospital, Eindhoven, Netherlands).
