# Supplementary material for: Treatment of locally advanced rectal cancer and synchronous liver metastases: multicentre comparison of two treatment strategies
Source: Br J Surg. 2023 Feb 23;110(9):1049–52. doi: 10.1093/bjs/znad013 (PMC10416702; doi:10.1093/bjs/znad013)
Supplement: znad013_Supplementary_Data [file znad013_supplementary_data.docx]

**Treatment of locally advanced rectal cancer and synchronous liver metastases: a multicentre comparison of two treatment strategies**

J.M. van Rees MD^1^*, M.F. Krul MD^2^*, N.F.M. Kok MD PhD^2^ , D.J. Grunhagen MD PhD^1^, E.N.D. Kok MD PhD^2^, P.M.H. Nierop MD PhD^1^, K. Havenga MD PhD^3^, H. Rutten MD PhD^4^, J.W.A. Burger MD PhD^4^, J.H.W. de Wilt MD PhD^5^, J. Hagendoorn MD PhD^6^, F.P. Peters MD PhD^7^, J. Buijsen MD PhD^8^, P.J. Tanis MD PhD^1,9^, C. Verhoef MD PhD^1^, K.F.D. Kuhlmann MD PhD^2^ , on behalf of the Dutch Stage IV Rectal Cancer Group.

* Both authors contributed equally to this manuscript
 **^1^** Department of Surgical Oncology, Erasmus MC Cancer Institute, Rotterdam, the Netherlands
^2^ Department of Surgical Oncology, The Netherlands Cancer Institute, Amsterdam, the Netherlands
^3^ Department of Surgery, University of Groningen, Groningen, the Netherlands
^4^ Department of Surgery, Catharina Hospital, Eindhoven, the Netherlands
^5^ Department of Surgery, Radboud University Medical Centre, Nijmegen, the Netherlands
^6^ Department of Surgery, University Medical Centre Utrecht, Utrecht, the Netherlands
^7^ Department of Radiation Oncology, Leiden University Medical Centre, Leiden, the Netherlands
^8^ ^8^ Department of Radiation Oncology (Maastro), GROW School for Oncology, Maastricht University Medical Centre+, Maastricht, The Netherlands
^9^ Department of Surgery, Amsterdam UMC, Cancer Centre Amsterdam, University of Amsterdam, Amsterdam, the Netherlands

**Collaborators (Dutch Stage IV Rectal Cancer Group)**

G.L. Beets, A.G.J. Aalbers, T. J. M. Ruers, C. B. H. A. Kobus, S. V. Siemons, C. Grootscholten, L.G.H. Dewit, J.G. van den Bergand, K.P. de Jong, G.A.P. Hospers, A. Karrenbeld, E.D. Geijsen, C.J.A. Punt, E. Gootjes, M.P.W. Intven, J.M.L. Roodhart , F. Holman, E. Kapiteijn, J. Melenhorst, J.S. Cnossen, G.J.M. Creemers

**Corresponding author**

J.M. van Rees, MD

Department of Surgical Oncology, Erasmus MC Cancer Institute, Rotterdam, the Netherlands

Dr. Molewaterplein 40, 3015 GD Rotterdam, the Netherlands

E-mail: [j.vanrees@erasmusmc.nl](mailto:j.vanrees@erasmusmc.nl)

<https://orcid.org/0000-0002-0194-8798>

**Supplementary Materials - Index**

| **Supplementary Methods** |  |
| --- | --- |
| Treatment schedules | *pag. 3* |
| Definitions and outcome measures | *pag. 3* |
| Statistics | *pag. 4* |
| **Supplementary Figures and Tables** |  |
| Table S1 | *pag. 5* |
| Table S2 | *pag. 6* |
| Figure S1 | *pag. 8* |
| **References** | *pag. 9* |
|  |  |

**Supplementary Methods**

This was a multicentre comparative cohort study in nine tertiary referral centres in the Netherlands, including patients with locally advanced rectal cancer and synchronous liver metastases with or without limited extrahepatic disease (potentially) amenable to local treatment. The Erasmus MC Cancer Institute exclusively used LFA in the period between January 2004 until December 2018, and the other eight Dutch centres applied the M1-schedule between January 2010 (initiation of M1-schedule) until December 2018. Patients with progressive disease or clinical deterioration before any local treatment was performed, were not included in this study. The study was approved by the medical ethics committees.

**Treatment schedules**

*Liver first approach*

In the Erasmus MC Cancer Institute, eligible patients were treated with the liver first approach.(1) Patients were first treated with systemic chemotherapy with or without bevacizumab and radiological tumour response was assessed after three cycles. If no disease-progression was observed, local treatment of the liver metastases was performed, sometimes preceded by some additional cycles of systemic therapy. Subsequent long-course radiotherapy was started, which consisted of 25 x 1.8-2 Gy with or without concomitant capecitabine.(3) Rectal surgery was planned after restaging with a thoracic- and abdominal CT scan and pelvic MRI, even when (near) complete response was observed. Eligible patients were planned for surgery 8-12 weeks after completion of neoadjuvant (chemo)radiotherapy.

*M1-schedule*

Eight centres in the Netherlands have been using the M1-schedule since 2010.(4) In this schedule, treatment started with short course radiotherapy, 5 x 5 Gy, followed by 3-6 cycles of systemic treatment, mostly consisting of doublet chemotherapy with or without bevacizumab. Subsequent local treatment of both tumour locations was planned after restaging with a thoracic- and abdominal CT scan and pelvic MRI. Sequence of local treatments differed, and either liver first, primary first or a synchronous resection was performed at the discretion of the treating MDT.

**Definitions and outcome measures**

Locally advanced rectal cancer was defined as a tumour with at least one of the following characteristics: tumour >5 cm; mesorectal fascia (MRF) ingrowth or ingrowth in adjacent organ on MRI (T4); N+ tumour, i.e. at least one lymph node >8 mm or 4 lymph nodes >5 mm on CT scan or magnetic resonance imaging (MRI).(1, 2) All patients were discussed in a multidisciplinary team (MDT) of liver- and colorectal surgeons, radiologists, radiation oncologists and medical oncologists before start of treatment and for response evaluation and re-staging in between treatment modalities. Patient-, tumour-, and treatment data were retrospectively obtained from electronic patient files. The study was approved by the medical ethics committees.

Main outcomes were overall survival (OS) and progression-free survival (PFS), independent of treatment completion. Other outcomes were clinical- and pathological complete response rates of the primary tumour, schedule completion rate, schedule duration, and complications. OS, PFS, and schedule duration were measured from the date of diagnosis of the liver metastases. The schedule was considered complete after resection of both tumour locations. Completion of the schedule was also considered to be achieved in case of a combination of surgery and a complete clinical response.

Progression was defined as either progression or recurrence of the local-, hepatic- or extrahepatic disease. Clinical complete response after neoadjuvant treatment of the primary tumour was achieved if pelvic MRI and/or endoscopy revealed no residual/recurrent local disease during three consecutive follow-up visits. Pathological complete response of the primary tumour was achieved when no vital tumour cells were found in the specimen. Complications were scored using the Clavien-Dindo (CD) surgical complication score. Only severe complications (≥ grade 3) were reported.(5)

**Statistics**

Continuous data were presented as medians and interquartile ranges (IQR). Categorical data were presented as numbers and percentages. Groups were compared using Chi-squared and Mann Whiney U test. Schedule duration and total length of stay were (separately) reported for patients who completed the schedule as well as for patients who did not complete the schedule. Missing or unknown categories were not included in statistical analyses. Kaplan Meier method was used to calculate OS and PFS. These were compared using the log-rank test. A P-value less than 0.05 was considered statistically significant. Statistical analyses were performed using R version 4.1.1 (<http://www.r-project.org>).

**Supplementary Figures and Tables**

**Supplementary Table 1: Treatment details**

|  |  | LFA (n=96) | M1 (n=164) | p-value |
| --- | --- | --- | --- | --- |
| Completion (%) | No | 24 (25%) | 31 (19%) | 0.245 |
|  | Yes | 72 (75%) | 133 (81%) |  |
| Stomy before/during scheme (%) | No | 78 (82%) | 117 (74%) | 0.140 |
|  | Yes | 17 (18%) | 41 (26%) |  |
| Moment of colostomy (%) | No colostomy | 39 (42%) | 76 (49%) | 0.016 |
|  | Before schedule | 8 (9%) | 31 (20%) |  |
|  | During schedule | 9 (10%) | 10 (6%) |  |
|  | At primary resection | 37 (40%) | 39 (25%) |  |
| Permanent colostomy at the end of treatment (%) | No colostomy | 39 (42%) | 76 (49%) | 0.299 |
|  | Colostomy | 54 (58%) | 80 (51%) |  |
| Number of systemic chemotherapy cycles (median [IQR]) | | 4.0 [3.0, 5.0] | 6.0 [3.0, 6.0] | 0.001 |
| Bevacizumab added to systemic therapy (%) |  | 24 (25%) | 110 (68%) | <0.001 |
| LM surgery type (%) | Hemihepatectomy | 22 (23%) | 34 (23%) | 0.008 |
|  | Segment/wedge resection | 33 (34%) | 65 (44%) |  |
|  | Ablation | 5 (5%) | 19 (13%) |  |
|  | Combined resection and ablation | 36 (38%) | 29 (20%) |  |
|  | *No LM resection** | *0* | *10* |  |
|  | *Missing** | *0* | *7* |  |
| LM surgery resection margins (%) | R0 | 83 (86%) | 106 (76%) | 0.214 |
|  | R1 | 8 (9%) | 18 (13%) |  |
|  | R2 | 0 (0%) | 2 (1%) |  |
|  | Ablation | 5 (5%) | 13 (9%) |  |
|  | *No LM resection** | *0* | *10* |  |
|  | *Missing** | *0* | *15* |  |
| LM pathological complete response (%) | Complete response | 10 (12%) | 18 (17%) | 0.357 |
|  | No complete response | 73 (88%) | 89 (83%) |  |
|  | *Ablation** | *5* | *13* |  |
|  | *No LM resection** | *0* | *10* |  |
|  | *Missing** | *8* | *34* |  |
| Rectal radiological response (%) | Complete response | 1 (2%) | 9 (7%) | 0.006 |
|  | Partial response | 48 (81%) | 122 (90%) |  |
|  | Progression of disease | 4 (7%) | 1 (1%) |  |
|  | Stable disease | 6 (10%) | 4 (3%) |  |
|  | *Missing** | *12* | *1* |  |
|  | *No MRI made* | *25* | *27* |  |
| Rectal surgery type (%) | APR | 20 (27%) | 37 (26%) | 0.005 |
|  | Exenteration | 8 (11%) | 2 (1%) |  |
|  | Hartmann | 0 (0%) | 8 (6%) |  |
|  | LAR | 45 (62%) | 89 (63%) |  |
|  | Local excision | 0 (0%) | 1 (1%) |  |
|  | *No primary resection** | *22* | *26* |  |
|  | *Missing** | *1* | *1* |  |
| Rectal surgery resection margins (%) | R0 | 62 (91%) | 111 (87%) | 0.427 |
|  | R1 | 6 (9%) | 16 (13%) |  |
|  | *No primary resection** | *22* | *26* |  |
|  | *Missing** | *6* | *11* |  |
| Rectal pathological complete response (%) | No complete response | 59 (91%) | 113 (88%) | 0.266 |
|  | Pathological complete response | 6 (9%) | 10 (8%) |  |
|  | Clinical complete response | 0 (0%) | 5 (4%) |  |
|  | *No primary resection** | *22* | *21* |  |
|  | *Missing** | *9* | *15* |  |
| Overall treatment duration (median [IQR]) | | 40.6 [31.1, 47.2] | 34.9 [28.1, 41.8] | 0.008 |
| Treatment duration if scheme completed (median [IQR]) | | 44.0 [39.5, 49.9] | 35.9 [29.5, 42.6] | <0.001 |
| Total length of stay if scheme completed (mean (SD)) | | 18.8 (8.9) | 18.0 (11.8) | 0.686 |

*Abbreviations: APR – abdominoperineal resection. IQR – interquartile range. LFA – liver first approach. LAR – low anterior resection. LM – liver metastases. M1 – M1-schedule. SD – standard deviation.** Not included in percentages

**Supplementary Table 2a: Complications according to the Clavien-Dindo classification and hospital stay after simultaneous resection**

|  |  | M1 (n=40) |
| --- | --- | --- |
| Hospital stay (median [IQR]) | | 11.5 [9.2, 20.2] |
| Simultaneous resection complications (%) | None | 14 (36%) |
|  | Grade 1 | 3 (8%) |
|  | Grade 2 | 9 (23%) |
|  | Grade 3 | 9 (23%) |
|  | Grade 4 | 3 (8%) |
|  | Grade 5 | 1 (3%) |
|  | *Missing** | *1* |
| Major complications (%) | Yes | 13 (33%) |

* Not included in percentages

**Supplementary Table 2b: Complications according to the Clavien-Dindo classification and hospital stay after liver treatment**

|  |  | LFA (n=96) | M1 (n=124) | p-value |
| --- | --- | --- | --- | --- |
| Hospital stay (median [IQR]) | | 6.5 [5.0, 8.0] | 7.0 [5.0, 10.0] | 0.468 |
| Liver resection complications (%) | None | 73 (78%) | 67 (64%) | 0.232 |
|  | Grade 1 | 6 (6%) | 7 (7%) |  |
|  | Grade 2 | 11 (12%) | 15 (14%) |  |
|  | Grade 3 | 2 (2%) | 9 (9%) |  |
|  | Grade 4 | 1 (1%) | 4 (4%) |  |
|  | Grade 5 | 1 (1%) | 3 (3%) |  |
|  | *Missing** | *2* | *4* |  |
|  | *No resection**† | *0* | *15* |  |
| Major complications (%) |  | 4 (4%) | 16 (15%) | 0.010 |

* Not included in percentages
† Patients with complete response of both tumour sites (n=1) and patients with primary resection only (n=14)

**Supplementary Table 2c: Complications according to the Clavien-Dindo classification and hospital stay after primary resection**

|  |  | LFA (n=96) | M1 (n=124) | p-value |
| --- | --- | --- | --- | --- |
| Hospital stay (median [IQR]) | | 9.0 [8.0, 11.8] | 9.0 [7.0, 13.8] | 0.481 |
| Primary resection complications (%) | None | 45 (62%) | 42 (49%) | 0.073 |
|  | Grade 1 | 2 (3%) | 10 (12%) |  |
|  | Grade 2 | 17 (23%) | 15 (18%) |  |
|  | Grade 3 | 6 (8%) | 15 (18%) |  |
|  | Grade 4 | 3 (4%) | 3 (4%) |  |
|  | Grade 5 | 0 (0%) | 0 (0%) |  |
|  | *Missing** | *1* | *13* |  |
|  | *No resection**† | *22* | *26* |  |
| Major complications (%) | Yes | 9 (12%) | 18 (21%) | 0.141 |

* Not included in percentages
† Patients with complete response of both tumour sites (n=1) and patients with liver resection only (n=25)
 *Abbreviations: LFA – liver first approach. M1 – M1-schedule. IQR – interquartile range.*

**Supplementary Figure 1: Flowchart selection criteria**


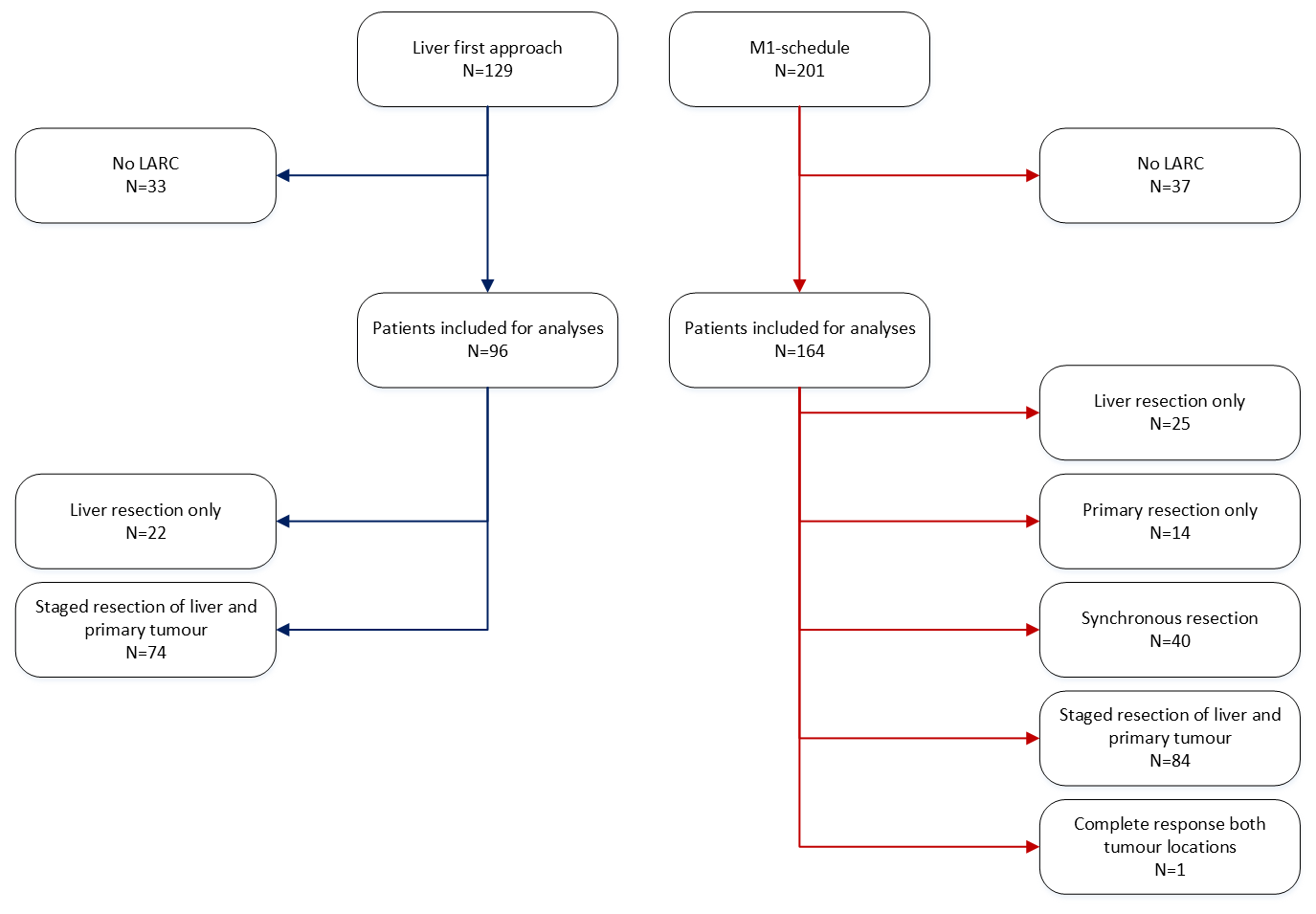


**References**

1. Verhoef C, van der Pool AE, Nuyttens JJ, Planting AS, Eggermont AM, de Wilt JH. The "liver-first approach" for patients with locally advanced rectal cancer and synchronous liver metastases. Dis Colon Rectum. 2009;52(1):23-30.

2. Ayez N, Burger JW, van der Pool AE, Eggermont AM, Grunhagen DJ, de Wilt JH, et al. Long-term results of the "liver first" approach in patients with locally advanced rectal cancer and synchronous liver metastases. Dis Colon Rectum. 2013;56(3):281-7.

3. de Bruin AFJ, Nuyttens JJ, Ferenschild FTJ, Planting AST, Verhoef C, de Wilt JHW. Preoperative chemoradiation with capecitabine in locally advanced rectal cancer. Netherlands Journal of Medicine. 2008;66(2):71-6.

4. van Dijk TH, Tamas K, Beukema JC, Beets GL, Gelderblom AJ, de Jong KP, et al. Evaluation of short-course radiotherapy followed by neoadjuvant bevacizumab, capecitabine, and oxaliplatin and subsequent radical surgical treatment in primary stage IV rectal cancer. Ann Oncol. 2013;24(7):1762-9.

5. Dindo D, Demartines N, Clavien PA. Classification of surgical complications: a new proposal with evaluation in a cohort of 6336 patients and results of a survey. Annals of surgery. 2004;240(2):205-13.
